# A longitudinal study of the turning points and trajectories of therapeutic relationship development in occupational and physical therapy

**DOI:** 10.1186/s12913-021-06095-y

**Published:** 2021-01-28

**Authors:** Ayana Horton, Gail Hebson, David Holman

**Affiliations:** 1 Brunel University, Uxbridge, UB8 3PH England; 2 Work and Equalities Institute, University of Manchester, Manchester, England; 3 Alliance Manchester Business School, University of Manchester, Manchester, England

**Keywords:** Therapeutic relationship, Turning point analysis, Therapeutic alliance, Helping relationship, Occupational therapy, Physical therapy

## Abstract

**Background:**

The importance of the therapeutic relationship is widely recognised across healthcare professions. Despite the importance of therapeutic relationships, there are significant gaps in the knowledge base on how these relationships develop. To address these gaps, this study explores relationship dynamics by identifying relational turning points and trajectories in therapeutic relationships between occupational therapists and physical therapists and their patients. The implications for how a focus on these relational aspects can enhance clinical practice will be discussed.

**Methods:**

Data collection was based on the Retrospective Interview Technique and consisted of two phases. In the first phase patients and therapists were asked to tell the story of their therapeutic relationship development and as part of this, identify the turning points that occurred. In the second phase, therapists-patient dyads were observed from their first interaction to their last to identify potential turning points and at the end of the relationship a participant verification interview was conducted with both dyadic partners individually. Template analysis was used to analyse the data.

**Results:**

Therapists identified 6 distinct categories of turning points; Progress Towards Goals, Set-backs in Progress Towards Goals, Interpersonal Affective Bonding with Patients, Interpersonal Problems with Patients, Positive Feedback, and Negative Feedback. Patients identified 5 categories of turning points; Progress Towards Goals, Set-backs in Progress Towards Goals, Interpersonal Affective Bonding with Therapists, Agreement with Therapist and Change in Treatment. These turning points varied regarding their impact on the trajectory of the therapeutic relationship. The trajectory patterns identified were stable, upward, downward, and multidirectional.

**Conclusion:**

This study makes an important contribution to our understanding of therapeutic relationship dynamics in the occupational and physical therapy context. The results expose the challenges that therapists and patients face in building high-quality therapeutic relationships, the diversity of therapeutic relationships, and how these relationships develop over time. This is the first study to use a turning point analysis in research on therapeutic relationships.

## Background

The therapeutic relationship refers to the interpersonal relationship between the healthcare professional and patient [1] and is also referred to in the research literature as a therapeutic alliance, a helping alliance or a working alliance. The importance of the therapeutic relationship is widely recognised across healthcare professions [e.g., nurses [1]; physicians [2]; psychotherapists [3]. Specifically, in the context of occupational therapy, researchers associate therapeutic success with the quality of the therapeutic relationship [4, 5]. In physical therapy, therapeutic relationships have been found to have a significant impact on measures of healthcare quality including clinical outcomes [6–8] and patient satisfaction [9].

The quality of therapeutic relationships can be understood in terms of the extent to which dyadic partners perceive the relationship as having particular attributes [10]. Common attributes include agreement on task, goals, trust, liking, interpersonal skills, responsiveness, and shared decision making. Relationships that rate high on these constructs are considered high-quality therapeutic relationships and those that rate low on these items are considered progressively lower quality relationships [10]. The quality of the therapeutic relationship has also been conceptualised as the level of authenticity and positive regard that dyadic partners display towards each other [11].

Given the importance of developing high-quality therapeutic relationships and avoiding low-quality therapeutic relationships, researchers have sought to understand how therapeutic relationships develop. Research on this topic in occupational therapy and physical therapy disciplines primarily focus on factors such as behaviours, shared understandings, and the presence of an interpersonal connection, that impact upon or promote therapeutic relationship development [12, 13]. Unfortunately, these studies overlook the relational events that cause these factors and the resulting trajectories of the relationship. Relational events cause actions, reactions, emotions, and thoughts that affect each dyadic partner’s ongoing perception of the relationship. In this way, these events constitute the dynamics of interaction within relationships [14].

Some researchers have sought to understand therapeutic relationship trajectories by charting the development of various component parts within the therapeutic relationship. For example, Wilson, Morse, and Penrod [15] conducted a study in caregiving relationships on how trust develops in therapeutic relationships, and Tickle-Degnen and Gavett [16] conducted a study in the context of speech and language therapy on how nonverbal behaviour develops in therapeutic relationships. In both studies, the early stages of the therapeutic relationship were characterised by both parties learning the rules of engagement. As the relationship progressed, following these rules of engagement led to decreased interpersonal boundaries, a sign of positive therapeutic relationship development. Unfortunately, these studies do not specify relational events that lead to decreased interpersonal boundaries.

Other researchers have also sought to understand the trajectory of therapeutic relationship development in terms of how the strength of the therapeutic relationship changes during the course of the relationship. Strength typically refers to how well the dyadic partners work together, how much they agree on goals, and how much they like each other [10]. The more they do these things, the stronger the therapeutic relationship. Researchers measure the strength of the therapeutic relationship using quantitative measures based on their theoretical conceptualisation of the therapeutic relationships and their clinical experience [17]. These measures are taken at different times during the therapeutic relationship to chart the trajectory of the relationship.

Using this approach, researchers have found that the therapeutic relationship in psychotherapy can have a stable, linear, or quadratic development pattern. A stable development pattern has little change across sessions. In a linear growth pattern, the strength of the relationship increases with each session. A U-shaped pattern is characterised with high affective bond in the first and last sessions and lower affective bond in the middle [18]. Lastly, a more ‘up and down’ pattern is characterised by incidences that happen in which negative feelings occur and are then resolved [19]. While these studies contribute to our understanding of therapeutic relationship trajectories, they are limited in that they do not discuss the events that cause or are associated with the participants ratings of the strength of the relationship.

Research on ruptures and repairs, and on critical incidents, highlights relational events that influence therapeutic relationship development. Ruptures and repairs are defined as tensions, conflicts, or misunderstandings and the eventual resolutions in relationships between patients and therapists, through which the relationship develops [20]. Ruptures are inevitable interpersonal events [21] and are common in high and low-quality therapeutic relationships [22]. Repairs are critical to achieve better patient outcomes [22]. According to Safran & Muran [20] there are two types of relationship ruptures; withdrawal ruptures, where the patient avoids the therapist and confrontation ruptures, where the patient expresses his anger in a direct manner. The main limitation with these studies is ruptures only focus on the negative events, despite the fact that the events that affect relationships may be positive or negative. Research on critical incidents in therapeutic relationships focuses on events that significantly influence the trajectory of the relationship and has been mostly conducted in nursing [23] and psychotherapy [24]. Unlike research on ruptures and repairs, research on critical incidents recognises both negative and positive events. Examples of critical incidents that have a positive effect on therapeutic relationship quality is a therapist using humour with a patient and the treatment yielding positive results. Examples of critical incidents that have a negative effect on therapeutic relationship quality include a therapist disregarding the patient’s desires or a misunderstanding between the patient and therapist. However, research on critical incidents in therapeutic relationships mostly only focuses on one event within a relationship, rather than each event that influences relationship development throughout the course of the relationship. Also, while these studies identify negative and positive critical incidents, they do not then link these incidents to the resulting quality of the relationship.

It must be noted that many of the studies on how therapeutic relationships develop are conducted in psychotherapeutic contexts. While there are many similarities between therapeutic relationships in psychotherapy and those in occupational and physical therapy, there are important differences. In particular, the therapeutic focus and process is different. In psychotherapy the focus is mainly on mental and behavioural functioning and the therapeutic process occurs through dialog. In occupational and physical therapy, the focus is on mental and physical functioning and the therapeutic process is comparatively more physical than in psychotherapy. This is important because psychotherapists may use themselves therapeutically within the therapeutic relationship differently than occupational and physical therapists. For this reason, the applicability of psychotherapy-based research on therapeutic relationships to the occupational therapy and physical therapy contexts is questionable [25].

One of the main limitations in research on therapeutic relationship development, particularly in research within occupational and physical therapy disciplines, is an inability to fully account for and examine relationship dynamics over time. This is due to the tendency of studies to investigate one point in time rather than throughout the duration of the relationship and a tendency not to link relational events to the quality of the relationship and the resulting trajectory. An alternative method that may address these limitations is a turning point analysis. Turning points are the most significant relational events that influence the trajectory of a relationship from each dyadic partners’ point of view [26]. A turning point analysis is an established research method used, particularly in relationship science studies, to understand how interpersonal relationships develop and change over time [27–29]. Using a turning point analysis, this study aims to understand what turning points do occupational/physical therapists and their patients experience in therapeutic relationships and how do these turning points influence therapeutic relationship trajectories.

## Method

### Study design

This research was conducted in two stages. In the first stage, semi-structured interviews were used with patients and therapists. They were asked to tell the story of a therapeutic relationship that they recently experienced and in doing so, highlight the turning points that occurred during the relationship and the resulting quality of the relationship. In the second stage, unstructured, non-participant observation of patient/therapist dyads during their interactions throughout their therapeutic relationships and semi-structured interviews with each dyadic partner individually at the end of the relationship were used to understand participants perception of turning points that occurred during therapeutic relationships and how the turning points affected the quality of the relationship. Ethical approval was granted from the University of Manchester and the National Health Service in the United Kingdom.

### Participants

A purposive sampling strategy was used to target occupational therapists, physical therapists, and their patients. Patients and therapists were eligible to participate if they could communicate in English, give informed consent, and were between the ages of 19 and 75. The upper and lower age limits were imposed during the ethical approval process to avoid populations that are considered vulnerable. It was important to have interviewees who could speak about current or recent therapeutic relationships to increase the likelihood that they would be able to remember the important details of the relationship. For this reason, therapists were eligible to participate if they were currently employed in a role that involved patient contact. Patients were eligible to participate if they were currently receiving therapy services. All participants gave written and verbal informed consent to participate in the study.

In the first stage, 22 therapists (13 occupational therapists, nine physical therapists) and 11 patients participated. The therapists ranged in age from their 20’s to their 60’s, and 19 were female and three were male. They worked in various specialty areas, such as musculoskeletal, accident and emergency, and neurology, and ranged in years of experience from one to 35 years. The patients ranged in age from their 20’s to their 60’s. Of the 11 patients, 6 were male and 5 were female. Eight of the patients were working with a physical therapist and the remaining three were working with an occupational therapist. Recruitment of interviewees ended at this point because the data reached a point at which themes were repeating and no new themes were emerging in line with the notion of theoretical saturation that originates in grounded theory [30].

In the second stage, 14 dyads were recruited. They included six dyads consisting of two therapists with three patients, and eight dyads consisting of eight patients and eight therapists. They were recruited from hand therapy clinics in London, United Kingdom. The therapists ranged in ages from their 20’s to their 40’s, had between two and 20 years of experience, and were all white British except two, who were Asian British. Three of the therapists were physical therapists, and the remaining five were occupational therapists. Seven of the therapists were female and one was male. The patients ranged in age from their 20’s to their 60’s. They were an ethnically mixed group, with half of them being white British and the other half being of various ethnic and national backgrounds. Five of the patients were male and three were female.

### Setting

Occupational therapists and physical therapists were recruited from a number of hospitals and clinics in the United Kingdom. They worked with patients to help rehabilitate them after illness or injury, and worked with clients in a clinic for more than one session, normally over several months. They worked with patients who had a wide range of diagnoses, including stroke, multiple sclerosis, carpel tunnel syndrome, and hip fractures. In the hand therapy clinics, therapists work with patients who are typically able-bodied except for their hand injury. Common diagnosis are finger fractures, Dupuytren’s contracture, carpel tunnel syndrome, and arthritis in the hand.

### Data collection

The data collection strategy was based on the Retrospective Interview Technique, which is a particularly interactive method that allows the interviewer and interviewee to make a visual representation of their relationship [31] and is often used in studies that seek to capture relational trajectories and turning points [26–28]. In the first stage, in-depth semi-structured interviews were conducted with therapists and patients. The interviews lasted for about an hour and were audio taped and transcribed. The interviews were all conducted by the first author and typically took place in private rooms on the hospital premises. On a couple of occasions, interviews with therapists were in public spaces (e.g., coffee shops and libraries) for the convenience of the interviewee.

Interviewees were asked to describe a current or recent therapeutic relationship from beginning to end in a storyline fashion. Therapists were asked to talk about a high-quality and a low-quality therapeutic relationship. Since it was assumed that patients would not have had multiple therapeutic relationships with therapists, patients were only asked to describe their current therapeutic relationship. As part of the description, they were asked to identify major turning points within the relationship. Turning points were defined as noteworthy moments in their partnership that alter the relationship either negatively or positively [32]. To ensure that interviewees understood what was meant by turning points, the term was fully explained and they were shown a graph of the turning points and relational trajectories within a romantic relationship borrowed from a study by Huston and colleagues (1981) [33]. Examples of the turning points listed in the romantic relationship example were the first big fight, the first sexual encounter, and the marriage proposal. Even though romantic relationships are different from therapeutic relationships, the interviewees could identify with the turning point examples and were able transfer this understanding to the context of the therapeutic relationship.

Participants were asked to help make a similar graph of the turning points that occurred in chronological order and the corresponding perceptions of relationship quality. We called the graph a theme-map. On the X axis, the turning points within the relationship were plotted along a timeline. On the Y axis, the quality of the therapeutic relationship was plotted on a scale of 0 to 10. Despite the quantitative nature of the scale, it was used to aid the discussion on qualitative changes in the relationship and the factors that influenced those changes. The theme-map helped the interviewees think in terms of the sequence of events and helped to ensure the precision and accuracy of meaning interpretation. Since it was completed in collaboration with interviewees, it enabled them to agree, disagree, and clarify the themes emanating from their story. In essence, it served as an immediate form of member checking. Similar visual aids have been used by other qualitative researchers to increase the trustworthiness of their interpretations [34].

In the second stage, data were collected using unstructured non-participant observation combined with a participant verification interview at the end of the relationship. Observation was used to enable the researcher to gain intimate knowledge of the relationship between the patient and therapist which could be used to ask informed questions during the participant verification interviews. Observation provides the most direct access to the social phenomenon that is being studied and enables the researchers to get an insider’s perspective [35]. It is an ideal way to collect rich data on behaviour and interpersonal interaction under the most natural circumstances [14, 36]. Observation is a useful way to understand the events that happen during social interactions and unfolding behavioural sequences [37].

Unstructured observation is not unstructured in a sense that it is unsystematic, rather it is unstructured in a sense that it does not follow an approach of strictly checking a list of predetermined behaviours or events, as is the case with structured observation [36]. Using this approach, the first author observed each dyad during their treatment sessions, from the first session where they initially met, to the last when the patient was discharged from therapy services. Therefore, the number of treatment sessions observed varied for each dyad but ranged from 2 to 9 sessions. The observation was done in person, with the observer seated in the clinic within hearing distance to the dyad being observed, and an audio recorder was used to record dialog. The observer was specifically looking to observe interactional dynamics that may be considered turning points by the participants.

The basis of the participant verification interview was the interview schedule and theme map from the first stage, however, for each dyad, the interview schedule was heavily augmented with questions and prompts informed by the data collected through observation. In this way the researcher could ask informed questions about interactional dynamics that occurred during the therapeutic relationship and verify her understanding with the participants perceptions. For example, if through observation of dyadic interactions, it appeared that a particular incident was a turning point in the relationship, the researcher would ask both dyadic partners about it during the participant verification interview. The theme-map was used in the same way during the participant verification interviews as it was used in the first stage.

Semi-structured interviews are an ideal complement to observation data collection methods. Observational methods enable understanding of the phenomenon to a deeper extent than just using information from interviews [35]. Using observation can address inconsistencies between what people say they do and what they actually do [38]. Since observation is an ongoing dynamic activity, it is more likely than interview data to provide evidence for how a social phenomenon evolves over time [36]. However, observation data, more than interview data, is subject to interpretation by the researcher [36]. For this reason, it is important for qualitative researchers to clarify and demonstrate how verification strategies are used to ensure trustworthiness [39].

All audio recorded data was transcribed in preparation for data analysis. The transcripts were vetted for accuracy by the first author by listening to the audio file while reading the transcript. Data collection and analysis was conducted in reiterative cycles, where each cycle of data analysis influenced the subsequent cycle of data collection and vice versa.

### Data analysis

The data were analysed using template analysis as described by King [40] and NVivo 10 software package. The data analysis began with the formulation of an initial template which consisted of codes based on prior research. In particular, Ohly and Schmitt’s [41] categories of affective events at work were used to derive the initial turning point codes for the template because they describe interpersonal events that may influence relationship trajectories at work. Relevant sections of each transcript were coded using King’s [40] description of the process as a guide. The constant comparative method [30] was used to find differences and similarities in each turning point. The rational for grouping and separating turning points was documented in a reflective journal. The process of identifying the types of relational trajectories was similar. Each cycle of data collection and analysis benefited from an increasingly more fine-tuned template and the researchers’ increasing level of familiarity with the data.

To code the trajectories of the therapeutic relationships, for each theme-map, a line was drawn connecting the participants ratings of relationship quality on a 0–10 scale at each turning point during the relationship. This provided a visual representation of the therapeutic relationship trajectories for each participant. Using a method similar to a study by [42], trajectories were coded according to visual similarity to relationship development trajectories identified in previous studies [18, 19, 27].

## Results

### Therapists perspectives on turning points

In telling their stories of high and low-quality therapeutic relationships, therapists identified 140 unique turning points that they believed influenced their own and their patient’s perception of relationship quality. These have been categorised as constructive and non-constructive to capture the way therapists and patients discussed them in relation to their goals of achieving high-quality relationships. Constructive turning points are those that help dyadic partners develop high-quality therapeutic relationships. Non-constructive turning points are those that hinder dyadic partners from achieving high-quality therapeutic relationships. The initial template was informed by Ohly and Schmitt’s [41] taxonomy of affective events at work. Using a constant comparative analysis revealed six distinct categories: progress towards goals, set-backs in progress towards goals, interpersonal problems with patients, positive feedback, interpersonal affective bonding with patients, and negative feedback (see Table 1).

**Table 1:** Therapists’ Perceptions of Constructive and Non-Constructive Turning Points

Constructive Turning Points	Non-constructive Turning Points
Progress Towards Goals	Set-backs in Progress Towards Goals
Interpersonal Affective Bonding with Patients	Interpersonal Problems with Patients
Positive Feedback	Negative Feedback

#### Progress towards goals

Progress towards goals was the most prevalent turning point in therapeutic relationships that therapists perceived as high-quality but was also present in therapeutic relationships that therapists described as low-quality. Turning points falling into this category concern the achievement of or progress towards rehabilitation goals in relation to the patient’s status or the provision of treatment. Progress towards goals regarding the patient’s status included the patient feeling better, decreased pain or improved functional status. It also included the patient becoming more empowered, compliant, being able to walk or go back to work, and finding ways to overcome difficulties. Progress towards goals regarding the provision of treatment included getting equipment in good time, smooth referrals to continuing care agencies, or finding funds for patient carers.

Therapists considered these turning points as conducive to their goals because they facilitated positive emotions in patients and therapists and had a positive effect on the quality of the relationship. For example, one therapist identified a turning point where her patient made some functional improvements. In response to these improvements, the therapist felt satisfaction which she then shared with her co-workers. She also felt increased fondness towards the patient and noted that this turning point led to increased rapport and trust between her and her patient.

“So basically, met him on his first day after surgery and he was quite a grumpy old man I think you could describe him as. And not engaged with therapy, not that, I guess not that trustful with what I was telling him. Because I was positive, possibly the same age as his grandchild and I think he probably thought, what does she know? And also, that I’m little, so things like getting someone out of bed and there’s a lack of trust for the person’s skill, is detrimental to that relationship…. when he could see that what we were doing was working, then that relationship got better and he got better subsequently. So, when that started to happen, he became more engaged and even when I wasn’t treating him, he would talk to me on the ward.”

(1-12-T) Therapist

#### Set-backs in Progress towards goals

Turning points in this category were mostly setbacks in patients’ progress or difficulties with the provision of services. The therapists described setbacks in the patient’s progress, such as the patient being unwell, experiencing increased pain, and the patient not being able to do as much as he or she expected. They also described setbacks, such as the patient experiencing a fall, embarrassing situations like a patient accidentally moving their bowels on the floor, and the patient’s family realising they cannot cope with the patient at home. Difficulties with the provision of services included ordered equipment arrived and was inappropriate, difficulties in getting required services funded, and difficulty in getting the patient a placement in a continuing care facility.

Turning points in this category are non-constructive to building positive therapeutic relationships because they typically provoked negative emotions, such as anger, frustration, and embarrassment and had a negative effect on the quality of the relationship. These turning points were described in therapists’ description of high and low-quality therapeutic relationships. For example, a therapist identified a turning point where the patient experienced increased pain because she was doing her exercises incorrectly.

“Next time I saw her, she comes in, the pain is worse, she’s frustrated, she’s upset, she’s depressed, she’s got fear for her future, she’s got a bit of embarrassment that things haven’t gone better, she’s hostile, a little bit hostile that things aren’t getting better and I looked at her exercise and she’d been doing everything wrong..”

(1-5-T) Therapist

The therapist reported that in response to this event he was frustrated, irritated, and on the verge of losing his temper. He noted that this turning point had a negative effect on his perception of the relationship quality.

#### Interpersonal problems with patients

This was the most prevalent type of turning point in therapists’ descriptions of low-quality relationships. However, one therapist described such a turning point in her description of a high-quality relationship. Turning points in this category includes disagreements and misunderstandings caused by the patient or the patient’s family not listening, being distrustful or lacking insight. This turning point category also includes situations where the patient is being noncompliant, manipulative, hostile, or just not participating in the rehabilitation process. These turning points were non-constructive to building positive therapeutic relationships. They typically had a negative impact on the therapists’ emotions, the relationship quality, and future interactions.

To illustrate, a therapist described a turning point with a difficult patient where she had just lifted him up, into his chair and he asked her to fetch his dressing gown. When the therapist retrieved it from the bag it was in, she noticed it was filthy and crawling with bugs. She was hesitant to give it to him, but he insisted. She took it outside to try and shake off the bugs, brought it back, and gave it to him. She then informed the Head Sister on the ward, who directed her to take the dressing gown off the ward to prevent an infestation. She had to take the dressing gown off him, despite his protests, and he was extremely cross. To regulate the patients’ emotions the therapist said she tried several things. First, she tried to get rid of the bugs and give the dressing gown to him. Then she tried to explain to him why they needed to get the dressing gown off the ward. She offered him drinks and lunch but that did not seem to calm him down. She even considered purchasing him an inexpensive dressing gown using her own money. Meanwhile, she hid her disgust in an effort to maintain a professional appearance. She reflected on the state of their therapeutic relationship after that incident and their ongoing interactions.

“…then today he’s almost going over the top and thinking that I’m killing him, and I’m giving him lots of problems because I won’t give him the dressing gown back… so, something really simple like that, now our therapeutic relationship is rock bottom.”

(1-13-T) Therapist

#### Positive feedback

Turning points in this category were mostly identified in therapists’ descriptions of high-quality relationships. Therapists received positive feedback from patients verbally, or in the form of a letter or a gift. Sometimes patients showed their appreciation in their behaviour, such as hugs or smiles. Other times patients showed appreciation by apologising for previous incidents. These turning points facilitated therapists to experience positive emotions, such as happiness, pride, and satisfaction and had a positive effect on their understanding of the relationship quality.

For example, a therapist described a situation where she visited one of her patients who had a terminal illness. The doctors did not expect him to live through the day because his oxygen levels were so low. His family were present and very emotional. During the therapist’s visit with the patient, she noticed that his oxygen mask was not fitted properly and corrected it. As a result, the patient recovered and lived another month and a half. When the patient could, he showed his appreciation for her help. The therapist appreciated his gratitude so much that she cried. She stated that she cried because it was natural, and he was crying too. She further explained that she cried because she understood what he had gone through and wanted to express that she cared. She perceived that the patient felt loved. The therapist believed this turning point had a positive effect on the quality of their therapeutic relationship.

#### Interpersonal affective bonding with patients

Turning points that fall in this category were identified in therapists’ descriptions of high-quality therapeutic relationships. This type of turning point was not identified in therapists’ descriptions of low-quality therapeutic relationships. Some of these turning points were just simple instances when patients and therapists were just talking, getting to know each other, and finding common ground. Therapist also described more pronounced turning points, such as instances where therapists provide emotional gifts to patients. Examples of which are a therapist making Christmas on the ward more festive to help a patient and his family enjoy their time on the ward, or a therapist going to visit a patient when he has been moved to a different ward. Events that fall into this turning point category help to build the patient’s and the therapist’s favourable impression of each other and their relationships. For example, one therapist described a turning point in a therapeutic relationship where her patient over heard the therapist advocating on the patient’s behalf, which in turn made the patient think more positively about the therapist. These turning points engendered positive emotions and positive perceptions of relationship quality.

One therapist described a situation where just taking the time to talk to his patient had a positive impact on the relationship quality.

“…we just sat down for about twenty minutes and just went through everything that was going on with him and explained to him about his fractures and his healing times and realised… he didn’t really know what went on. So, it was actually giving him a bit of an update about what went on and how he will get on. And his key question after that is, will I play golf? And probably in my partially optimistic way I said, in an ideal world there’s no reason why you can’t get back to playing golf. And then I think that started to, he knew a little bit more and he was like, oh thank you, you’re the first person to actually talk to me and tell me what’s going on and how long things will take.”

(1-10-T) therapist

#### Negative feedback

Turning points involving patients or their family giving negative feedback were described in high and low-quality relationships. These are non-constructive turning points because they provoke negative emotions and have a negative impact on the quality of the relationship. Patients gave negative feedback mainly regarding pain that they experienced due to their therapy. Patients also gave formal and informal complaints to management and other healthcare professionals regarding their dissatisfaction or disagreement with their therapist. For example, one patient’s family complained to a doctor to try to get the doctor to encourage the therapist to reconsider a decision she made with which the patient and the patient’s family disagreed. This made the therapist very angry and had a negative effect on her perception of the quality of the relationship.

Another therapist recalled a turning point where she ordered a bed for her patient who was being discharged home. When the patient got home and received the bed, he found that the bed did not meet his expectations. Although the therapist worked hard to find a solution to the problem, the family ultimately issued a formal complaint. The therapist reported that she was ‘livid’.

“…I said to my manager, ‘I bent over backwards to help this family, and this is what I get, you know’.”

(1-14-T) Therapist

While the majority of relationships had constructive and non-constructive turning points, high-quality relationships had more constructive turning points and low-quality relationships had more non-constructive turning points. In high-quality relationships, the most prevalent turning points were progress towards goals. The 2 s most prevalent types of turning points were set-backs in progress towards goals and affective bond building. The most prevalent turning points in low-quality relationships was interpersonal problems with patients. In contrast, this turning point was described only once in a high-quality relationship. The second most prevalent type of turning points in low-quality relationships was set-backs in progress towards goals. Interpersonal affective bond building was prevalent in high-quality relationships but was not described in relationships perceived as low-quality.

### Patients’ perspectives on turning points

Patients in the first and second stage of the research process described their current therapeutic relationships with their therapists. While some did not identify any events that they thought were significant enough to be considered turning points, other patients identified turning points that they believed influenced their own and their therapist’s emotions and perceptions of relationship quality. In total, 59 unique constructive and non-constructive turning points were identified. Using the turning points found in the therapists’ descriptions, and the patient nominated therapeutic relationship building critical incidents taxonomy developed by [24] to inform the initial template, five categories of turning points from the patients’ point of view were identified. The categories include: progress towards goals, set-backs in progress towards goals, interpersonal affective bonding with therapists, agreement with therapists and changes in treatment (see Table 2).

**Table 2:** Patients’ Perceptions of Constructive and Non-Constructive Turning Points

Constructive Turning Points	Non-constructive Turning Points
Progress Towards Goals	Set-backs in Progress Towards Goals
Interpersonal Affective Bonding with Therapists	
Agreement with Therapist	
Change in Treatment^a^	

^a^indicates that turning point may be either a constructive or non-constructive turning point

#### Progress towards goals

The most prevalent turning point category among patients was progress towards goals. The turning points in this category included the therapist quickly found and fixed the patients problem, pain decreased, functional abilities improved, and the patient received needed equipment quickly. This turning point category is similar to the therapist turning point category, Progress Towards Goals.

These turning points were conducive to patients’ goals of developing and maintaining positive therapeutic relationships because they understood them to have a positive effect on their own and their therapist’s emotions. They also understood these turning points to have a positive impact on the quality of the relationship. In fact, they were the most impactful in terms of patient’s perception of how much they changed the quality of the relationship. For example, one patient described a turning point where the therapist used a treatment that was unexpectedly effective.

“He [referring to the therapist] pulled my leg. Literally. And I didn’t even know it was going to happen… that was a bit of a shock. It took the words away from me…it didn’t hurt but it was, I wasn’t expecting it, so… But then it turned to even more surprise when I could see the results of what that had done. So, one pull on my leg and it’s kind of rectified the problem a little bit. So, I was very surprised that that would even have an effect.”

(1-8-P) patient

#### Set-backs in Progress towards goals

Patients described a number of turning points that can be categorised as Set-backs in Progress Towards Goals. Turning points in this category include experiencing treatments that caused pain, lack of progress, experiencing a decline in function associated with the treatment, and being provided with the wrong information. This turning point category is similar to the therapists’ turning point category of the same name. Unsurprisingly, turning points in this category tended to have a negative effect on the patient’s emotions and perception of relationship quality and therefore, were non-constructive to their therapeutic relationship goals. For example, one patient talked about a turning point that occurred when she received the wrong information which led her to go to her therapy session on the wrong day.

“I was annoyed… I had got myself all worked up and, so I was annoyed… I just said, ‘this is wasting my whole day from work, you know. Why can’t you get it right?’ You know, so I think, you know my whole dialogue with her was of a negative nature.”

(1-7-P) patient

#### Interpersonal affective bonding with therapists

This was another prevalent turning point category that is constructive to patients’ therapeutic relationship goals. This turning point category involves instances that build the affective bond between patients and their therapists. Patients described turning points in this category as superficial as just talking to and laughing with to their therapist, to more intimate events, such as a therapist helping a patient with his research project in her personal time. Through getting to know and finding common ground with their therapists, patients experienced positive emotions and developed a positive view of their therapist and the relationship as a whole. One patient described how she felt when, through conversation she and her therapist discovered they previously worked for the same organisation.

“I think there’s a sense of identification because we’ve talked about the negative points as well as the positive points (of working for the organisation). So, understanding what the other one is saying. Because it’s a sense of, well, yes, I know exactly how that feels because I’ve worked with them, and I know what that feels like because I have experienced that. So, there’s a bit of identification I suppose… and understanding. As I’ve got to know him, I’ve got to like him more.”

(1-3-P) patient

Another patient spoke about an important turning point in her therapeutic relationship where she disclosed personal information to her therapist and the therapist responded compassionately. She explained this was a turning point because her therapist’s response made her think that the therapist was “lovely”.

#### Agreement with therapist

Patients described agreements with their therapists as important turning points in their therapeutic relationships. In this category, patients described instances where their therapist helped them make important decisions or confirmed their thoughts or feelings. For example, one patient described a turning point in his relationship with his therapist where his therapist helped him make a decision with which he was struggling.

“…she told me I think you have made the right decision, but I didn’t make the decision. She helped me make the decision. I was able to make the decision through her and this is important communication... I felt that I could express myself freely (with the therapist).”

(2-5-P) patient

These instances may be noteworthy in patients’ perceptions of their relationships because the therapist’s agreement can serve to allay the patients fears and anxieties. For example, a patient described an interaction with her therapist where the therapist confirmed the patient was not ready to return to work.

“I was nervous about not getting back to work because I was worried about my job and how they would feel with me being off work for so long…I didn’t know if I was imagining, you know, putting off going back to work, so it made me feel better that someone who knows what they were talking about was saying that I wasn’t ready for work.”

(2-7-P) patient

#### Changes in treatment

Finally, patients described turning points that can be categorised as changes in treatment which can have either a negative or a positive effect on the relationship. Patients described feeling anxiety and fear prior to the first treatment session due to not knowing what to expect. They also described feeling anxiety and hopeful when changes were made to the treatment regime and when they were preparing for discharge. For example, a patient described how he felt when his therapy changed to focus on scar massage, a type of therapy that required his therapist to touch him more than was the case up to that point.

“Having her work on my hand was incredibly positive. I felt that was just going to sort everything out because I felt very positively about her. It’s like how could the bad stuff resist being driven away by her (the therapist) care.”

(1-9-P) patient

The same patient described a subsequent turning point that occurred when his therapist told him that she would no longer be working with him on a 1 to 1 basis due to an administrative change. He said he was “heartbroken” by the news and described how he addressed his emotions after the therapy session.

“That was just before Easter, I think, so I had an Easter that, in a tiny and pitiful way, mimicked the Christian Easter because I had a day of despair on the Friday; then on Saturday I stopped and thought about stuff; Sunday I thought about stuff and then on Monday I rose again. I think I kind of had the opportunity to digest what she had told me and think about how things should work. And decide what I was going to, you know, recognise that I had to do more. If she wasn’t going to be there every week, I needed to make sure I was on the ball.”

(1-9-P) patient

### Congruency of patients’ and therapists’ perceptions of turning points

In the second stage of data collection, patient and therapist dyads were observed during their interactions and then interviewed about the relationship. This provided a unique opportunity to gain insight on how dyadic partners perceived the same relationship events and turning points. When describing turning points, patients and therapists tended to identify corresponding turning points rather than the exact same turning points.

For example, C (2–5-T), the therapist, and A (2–5-P), the patient had a therapeutic relationship that spanned three treatment sessions. Patient A said that a turning point in their relationship happened when he found out that his therapist’s name is C. He said that the name brought back fond memories; it was the name of an old girlfriend of his, from many years ago, when he was a young man. Therapist C identified the first turning point as when she perceived that Patient A turned up to the first session prepared to listen to her. On the surface these turning points may seem to be unrelated. However, they may represent two sides of the same coin in that they represent both the therapist’s and patient’s perception of a positive first meeting in their own words.

In the second treatment session, Therapist C identified a turning point where Patient A brought in a store-bought splint that he was using instead of the splint that she made for him during the first treatment session. The therapist explained to the patient why the store-bought splint was not appropriate for his needs. She explained that if he was having problems with the splint she made for him, he should have come to her, so she could fix it. C said she felt annoyed with herself because she worried that she did not make it clear to A that he could bring the splint back to her to fix. She also felt a bit irritated and demotivated because she felt that he just discarded the work she had done. She perceived that the patient may have been upset by her response to his store-bought splint. She joked with him, saying ‘you’re going to put me out of business.’ Patient A accepted her feedback and agreed to use the custom-made splint. Significantly, he did not identify this incident as a turning point.

In the third and final treatment session, the therapists helped the patient come to a decision regarding a surgical procedure that the patient was worried about. Patient A considered this an important turning point in their relationship and stated that he felt relieved by the decision that the therapist helped him to make. The patient gloated about her skills as a therapist and proclaimed that she deserved a promotion. Therapist C, however, did not identify helping A come to a decision as a turning point, instead her perception of the turning point was that A seemed to be satisfied with the service he received. While A did not consider his bringing in a store-bought splint a turning point, it is clear that he had some awareness of the significance of the incident because during the participant verification interview he said that part of the reason that he said she deserved a promotion is because she previously joked that he was trying to put her out of business.

### Turning points and therapeutic relationship trajectories

The coding process revealed four distinct relational trajectories which we labelled as an upward trend, a downward trend, a multidirectional trend and a stable trend. The upward trend is characterised by successive improvements in the relationship quality while the downward trend is characterised by successive decreases in the quality of the relationship. The multidirectional trend is characterised with increases and decreases in therapeutic relationship quality. Lastly, the stable trend is characterised by no change in the quality of the therapeutic relationship.

Therapists’ perceptions of increases in relationship quality corresponded to constructive turning points and decreases in perceived relationship quality corresponded with non-constructive turning points. Relationships that were perceived as high-quality typically had an upward trend and relationships that were perceived as low-quality tend to have a downward trend. This is not surprising since, as stated above, high-quality relationships had more constructive turning points and low-quality relationships had more non-constructive turning points. Interestingly, relationships that were perceived as high-quality typically had a final turning point that had a positive effect on perceived relationship quality. Similarly, relationships that were perceived as low-quality tended to have a final turning point that had a negative effect on perceived relationship quality.

**Fig. 1:**
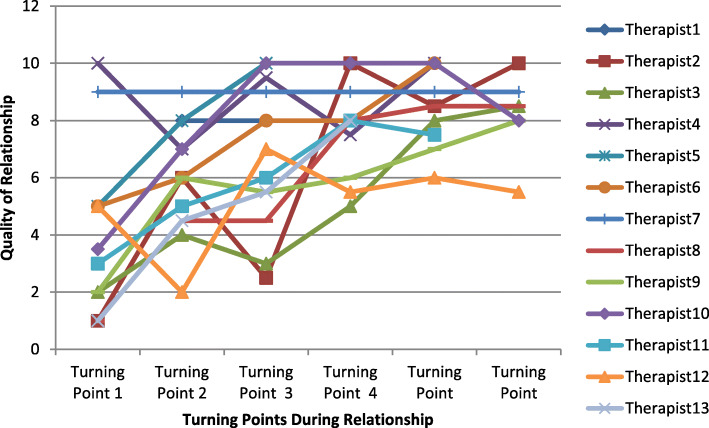
Graph of Therapists’ Perception of Therapeutic Relationship Quality at Each Turning Point in High-Quality Therapeutic Relationships

**Fig. 2:**
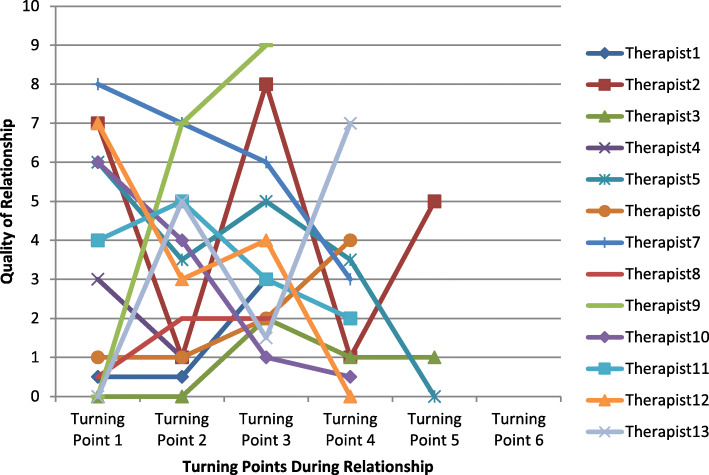
Graph of Therapists’ Perception of Therapeutic Relationship Quality at Each Turning Point in Low-Quality Therapeutic Relationships

Most therapeutic relationship trajectories had a multidirectional pattern with few trajectories displaying a straight upward, downward, or stable pattern. Since constructive turning points typically had a positive effect on therapist’s perception of relation quality and non-constructive turning points typically had a negative effect on therapist’s perception of relation quality, the multidirectional pattern is illustrative of the fact that most high and low-quality therapeutic relationships feature both constructive and non-constructive turning points. Figure 1 is a graph of therapists’ quality ratings at turning points in relationships perceived to be of high-quality. Figure 2 is a graph of therapists’ quality ratings at turning points in relationships perceived to be of low-quality. The data in the graphs are from the first stage of data collection where therapists were asked to describe a high and low-quality therapeutic relationship.

## Discussion

This study sought to understand what turning points therapists and patients experience during therapeutic relationships and how these turning points influence therapeutic relationship trajectories. Turning point analysis has long been used in research on personal relationships to understand relationships dynamics over time [26]. This is the first study to explore therapeutic relationship dynamics by identifying the turning points that occur over time from both dyadic partner’s point of view. The turning point categories are congruent with the critical incidents and ruptures and repairs identified in previous studies [22–24]. However, the findings of this study extend our understanding of relational events in therapeutic relationships in that it highlights the positive and negative relational events that occur during therapeutic relationships and links these events to the resulting quality of the relationship.

Although interpersonal events are considered an important aspect of therapeutic relationships [43], occupational therapy and physical therapy research on events that are influential in therapeutic relationship development is lacking. Instead, research on therapeutic relationships in occupational therapy and physical therapy contexts has mainly focused on understanding essential aspects of therapeutic relationships and only imply the importance of certain events in therapeutic relationship development. For example, Miciak and colleagues (2018) [44] identified therapists and patients being themselves or being genuine during interactions as a necessary condition for building therapeutic relationships. Similarly, Finaret and Shor [45] concluded that a necessary element involved in therapeutic relationship development is instances where the professional/personal boundary shifts to some degree towards the personal end of the spectrum. Shifting this boundary towards the personal end of the spectrum was aided by open communication, careful self-disclosure, and informal work settings, for example working in the client’s home [45]. These two studies imply that instances where dyadic partners are being transparent or disclosing information about themselves may be important interpersonal events within therapeutic relationship development. The findings of this study extend these studies by taking a more direct and in-depth approach to understanding the characteristics of turning points and how they influence therapists’ and patients’ perceptions of relationship quality. This is important because a better understanding of the nature of these influential events within therapeutic relationships can lead to a better understanding of how therapists can navigate such events successfully and therapists’ ability to resolving conflict within therapeutic relationships has an important impact on patient outcomes [46].

Regarding therapeutic relationship trajectories, constructive turning points tend to encourage people to feel more positively about the relationship and compel each individual to act accordingly. Similarly, non-constructive turning points tend to encourage people to appraise their relationship more negatively and to act accordingly [47]. By charting changes in dyadic partners’ perception of relationship quality at each turning point throughout the duration of the therapeutic relationship, this research has identified four distinct relational trajectories which we labelled as a multidirectional trend, a downward trend, an upward trend, and a stable trend. These trends were similar to therapeutic relationship trajectory patterns found in previous studies [18, 19]. However, unlike these previous studies, no evidence of U-shaped trajectory patterns was found. In psychotherapy research, a U-shaped relational trajectory has been associated with better outcomes than the other trajectory patterns [48]. This maybe because as psychotherapy progresses, the challenges arise creates a tension. Once the tension has been resolved, the relationship is restored or enhanced [49]. Since, psychotherapy is a talking therapy, whereas occupational and physical therapy are comparatively more physical, it may be that creating and resolving tensions is more important and prevalent in psychotherapy therapeutic relationships than occupational and physical therapy therapeutic relationships.

Understanding the turning point categories may have practical value to therapists in helping them to improve their practice. Therapists developing an awareness of turning points will help them be more in-tune to situations that lead to relational changes. This increased sensitivity can enable therapists to give the necessary attention to these situations so that they can positively influence the relationship’s trajectory. Understanding turning points will also provide a focal point when reflecting on practice. Therapists can reflect on how they responded to turning points and their patient’s response to gain insight on how they can better respond to such turning points in the future.

The turning point categories can also be used to help organizations and supervisors offer more realistic training opportunities. Therapists can use the non-constructive turning points in role playing exercises to practice their ability to navigate such situations. They can also use knowledge of constructive turning points to devise strategies to facilitate such turning points. In addition, given that all therapeutic relationships are different and develop in their own way, training programs should empower therapists to make decisions on the best way to interact with each patient, rather than attempting to standardise the way they interact with patients.

Future research on the topic should focus on whether therapists working in different therapeutic specialty areas tend to experience different types of turning points. Also, since both high- and low-quality therapeutic relationships had both constructive and non-constructive turning points, it is clear that the presence of non-constructive turning points does not guarantee low-quality therapeutic relationships. Likewise, the presence of constructive turning points does not guarantee high-quality therapeutic relationships. Future research should focus on what happens at those turning points that has a negative and positive influence on the trajectory of the relationship. Lastly, only therapists were asked to describe the turning points within high- and low-quality therapeutic relationships because it was assumed that most patients would not have experiences of numerous therapeutic relationships from which to choose. It would be interesting to research the turning points in high- and low-quality therapeutic relationships from the point of view of ‘professional patients’ or patients who have had multiple therapeutic relationships.

As with all studies, there are limitations that must be acknowledged. While semi-structured interviews are a useful way to access participants perceptions, the information gained may be limited by participants memory, understanding of the topic, or their willingness to disclose information. To address this issue in the first stage of data collection therapists were asked to discuss two recent therapeutic relationships and patients were asked to discuss their current therapeutic relationship. In the second stage of data collection the use of observation combined with participant verification interviews also mitigated some of these limitations since the researcher could ask questions based on her observations that might jog participants memory or compensate for any deficits in their understanding. However, using observation introduced additional limitations since participants may act differently when being watched. Also, the observation was done by one person, the first author, so this may have limited the depth and range of observations made, however the participant verification process allowed the observer to check whether her observations and interpretations matched those of the dyadic partners.

These limitations may have impacted the data collected, and therefore the range of findings and conclusions. Despite these limitations, semi-structured interviews and non-participant observation was used because these methods are ideal for exploring peoples’ perceptions and how people interact. However numerous strategies were used to limit their impact including methodological triangulation and the use of theme-maps as an immediate form of member checking. Future studies could video record therapeutic sessions and then have more than one researcher observe patient/therapist interactions using the recordings. This would limit the impact of the researcher on behaviour of those being observed.

Another limitation is the interview format may have encouraged participants to identify turning points to satisfy the interviewer. While, this did not seem to be the case as some participants did not identify major turning points, future studies could avoid this issue by giving participants an option to say there were no turning points in their relationship. Lastly, there was a limited sample size. For this reason, the transferability of the results are limited to the specific context in which the research was conducted.

## Conclusion

This study sought to understand how the dynamics of therapeutic relationships development by identifying relational turning points and trajectories in therapeutic relationships between occupational therapists and physical therapists and their patients. The results expose the challenges that therapists and patients face in building high-quality therapeutic relationships, the diversity of therapeutic relationships, and how these relationships develop over time. This is the first study to use a turning point analysis in research on therapeutic relationships.

## Data Availability

All data generated or analysed during this study are available from the corresponding author upon reasonable request.
